# Impact of the COVID-19 pandemic on some modifiable risk factors of dementia in an aging, rural Indian population

**DOI:** 10.3389/fpsyt.2023.954557

**Published:** 2023-05-19

**Authors:** Jonas S. Sundarakumar, Abhishek L. Mensegere, Palash K. Malo, Vijayalakshmi Ravindranath, on behalf of SANSCOG Collaborators

**Affiliations:** Centre for Brain Research, Indian Institute of Science, Bangalore, India

**Keywords:** pandemic (COVID-19), COVID-19, rural India, dementia, elderly, cardiovascular risk factors

## Abstract

**Introduction:**

The impact of the COVID-19 pandemic and associated lockdowns is likely to have caused adverse changes in lifestyle-related/cardiovascular risk factors and other such modifiable risk factors of dementia. We aimed to examine the pandemic’s impact on some modifiable risk factors of dementia among rural Indians belonging to a large, prospective aging cohort—Srinivaspura Aging, NeuoSenescence, and COGnition (SANSCOG).

**Methods:**

This was a cross-sectional study among adults aged ≥ 45 years (*n* = 3,148; 1,492 males and 1,656 females) residing in the villages of Srinivaspura in Karnataka state, India. SANSCOG study data (clinical and biochemical assessments) of these participants were obtained from three distinct periods: (i) the “pre-COVID period”—before India’s nationwide lockdown on 24 March 2020, (ii) the “COVID period”—during the first and second waves of the pandemic, wherein the social restrictions were prominent (25 March 2020 to 30 September 2021), and (iii) the “post-COVID period”—after easing of restrictions (from 1 October 2021 onward). Proportions of participants with diabetes, hypertension, obesity, dyslipidemia (diagnosed using standard criteria), and depression (diagnosed using the Geriatric Depression Scale) were compared between the above three periods.

**Results:**

The odds of having obesity, abnormal triglycerides, and depression among individuals in the COVID period were 1.42 times, 1.38 times, and 2.65 times more than the odds in the pre-COVID period, respectively. The odds of having hypertension, obesity, abnormal total cholesterol, abnormal triglycerides, abnormal LDL, and depression among individuals in the post-COVID period were 1.27 times, 1.32 times, 1.58 times, 1.95, 1.23, and 3.05 times more than the odds in the pre-COVID period, respectively. The odds of diabetes did not differ between any of the three periods.

**Discussion:**

We found significantly higher odds of some of the studied risk factors in the COVID and post-COVID periods compared to the pre-COVID period, suggesting that the pandemic adversely impacted the physical and psychological health of this marginalized, rural Indian population. We call for urgent public health measures, such as multimodal, lifestyle-based, and psychosocial interventions, to mitigate this negative impact and reduce the future risk of dementia.

## 1. Introduction

The COVID-19 pandemic resulted in a global public health emergency and upended the lives of millions of persons worldwide. During the first wave of the pandemic, India underwent one of the world’s biggest nationwide lockdowns from 25 March to 31 May 2020 (1). These lockdowns strictly prohibited individuals from leaving their homes except in emergencies, shut down public transport systems, and closed all offices, businesses, and institutions, barring essential services. After the total lockdown period, there were phased reductions in restrictions that extended till the onset of the second wave.

The second wave that started in February 2021 was associated with relatively milder, state-wise lockdowns, implemented entirely or partially according to the prevailing situations in localized areas. In Karnataka (where our study was conducted), containment measures, such as movement and transport restrictions, including night curfews, continued till May/June 2021, after which there were relaxations in inter-state, intra-state transport, economic activities, and work-related activities. By the end of October 2021, social gatherings, including cinema halls, auditoriums, and similar places, were permitted to function at 100% occupancy (2). The third wave in early 2022 did not entail any prominent lockdowns, and control measures were sporadic. So, it can be said that significant social restrictions in Karnataka due to the COVID-19 pandemic lasted till the end of September 2021. Though these public health measures were undoubtedly essential to control the disease spread and reduce mortality, the stringent lockdowns, particularly during the first wave, had prominent socioeconomic and health-related implications (3–7).

The pandemic and associated restrictions also resulted in substantial lifestyle changes. Studies worldwide have reported reduced physical activity (8, 9), altered dietary behaviors (10–12), and diminished psychosocial functioning (13, 14). Similar changes were also observed among Indians, with studies from different parts of the country reporting an increase in unhealthy eating habits, decreased physical activity, and weight gain (10, 15–17). Thus, it is possible that the pandemic had an impact on the prevalence of lifestyle-related disorders, such as diabetes, hypertension, dyslipidemia, obesity, etc. Further, it overburdened India’s healthcare system, and therefore, people’s access to routine healthcare was considerably hindered (18–21). These challenges, in turn, adversely affected the management of individuals with chronic diseases (22, 23). In addition, the pandemic led to increased stress, and some studies from India have revealed a negative impact on psychological health (14, 24–26).

Though the impact of the pandemic was wide-ranging, vulnerable populations, such as older adults, had disproportionately higher adverse effects. This impact was seen not only in direct effects, such as increased susceptibility to infection, more severe symptoms, and worse outcomes, including higher mortality (27), but also in indirect effects, such as social isolation, disruption in routine lifestyle, and poor access to healthcare (28, 29).

India’s older population is growing rapidly and will reach 353 million by 2050 (30). This demographic transition will be accompanied by an increase in the prevalence of dementia. Therefore, it is essential to examine the adverse effects of the COVID-19 pandemic on the aging Indian population, specifically on lifestyle-related disorders, such as diabetes, hypertension, obesity, dyslipidemia, and psychological disorders, such as depression, since they are recognized modifiable risk factors of dementia (31–33). As such, the prevalence of the above risk factors is generally on the rise among Indians owing to rapid urbanization and its associated lifestyle changes. The pandemic’s potential adverse effects could precipitate this situation, which, in turn, would further worsen the burden of dementia in the country.

We are conducting a large-scale, population-based, prospective cohort study on aging in rural Indians, namely Srinivaspura Aging, NeuoSenescence, and COGnition (SANSCOG) study (34). This cohort study aims to understand the differential trajectories of aging and identify risk and protective factors for dementia. SANSCOG cohort includes cognitively healthy aging individuals who undergo multimodal (clinical, cognitive, biochemical, genetic, and neuroimaging) assessments and are periodically followed up over a long term (at least 10 years).

SANSCOG cohort participants, who hail from a rural area in Karnataka in southern India, were substantially impacted by both waves of the pandemic. The first wave lockdowns entailed a prominently adverse financial impact for our predominantly agriculture-dependent participants due to hampering harvest, transport, and sale of their farm produce (35, 36). During the second wave, there was a severe healthcare crisis when the infections rapidly spread among these rural areas. The already fragile rural healthcare infrastructure in these areas was overwhelmed with the massive load of COVID cases. Therefore, the pandemic considerably disrupted our study participants’ everyday lives and significantly impacted their lifestyles.

In the current study, we aimed to examine the impact of the COVID-19 pandemic on specific modifiable risk factors of dementia, namely diabetes, hypertension, obesity, dyslipidemia, and depression in aging Indians from the SANSCOG study cohort. We hypothesized that there would be an increase in the prevalence of one or more of the risk factors mentioned above in the post-COVID period compared to the pre-COVID period. However, since these risk factors are potentially modifiable, prompt identification and appropriate mitigative measures can be put in place, which is why this study is important in the purview of dementia risk reduction.

## 2. Methods

### 2.1. Study design

A cross-sectional study design was employed for this study, wherein baseline clinical and biochemical assessment data of SANSCOG cohort participants were utilized.

### 2.2. Setting

SANSCOG cohort study is being conducted in a community-based setting in the villages of Srinivaspura ‘taluk’ (sub-district) in Kolar district, Karnataka state, India.

### 2.3. Recruitment

SANSCOG cohort study employs an area sampling strategy, a non-probabilistic sampling technique, wherein eligible and consenting participants are recruited from the villages of Srinivaspura.

### 2.4. Participants

SANSCOG cohort recruits rural-dwelling, cognitively healthy individuals (males and females) aged 45 years and above from the Srinivaspura area. Individuals with a known diagnosis of dementia (additional dementia screening was also done at the community before recruitment), psychosis, bipolar disorder, substance dependence (except nicotine), and any severe medical illness or significant hearing or vision impairment likely to limit the study evaluation are excluded. Further details of SANSCOG study recruitment and assessments are published elsewhere (34).

This study included 3,148 SANSCOG cohort participants (males: 1492, females: 1656) who had completed their baseline clinical and blood biochemical assessments. These participants underwent their assessments from 1 January 2018 to 30 April 2022. They were then divided into three mutually exclusive groups based on the timing of their baseline assessment. The three groups of participants were as follows:

- Participants who had completed their baseline assessments in the “pre-COVID period” (1 January 2018 to 24 March 2020).
- Participants who had completed their baseline assessments in the “COVID period” (25 March 2020 to 30 September 2021).
- Participants who had completed their baseline assessments in the “post-COVID period” (1 October 2021 to 30 April 2022).

### 2.5. Ethics clearance and informed consent

SANSCOG study has obtained ethics clearance from the Institutional Ethics Committee (Institutional Review Board) of the Centre for Brain Research, Indian Institute of Science, Bangalore, India. Written, informed consent was obtained from all participants before recruitment.

### 2.6. Measurements

- *Clinical assessments*: Clinical assessments were conducted by trained clinicians or nurses, and data were collected using handheld digital devices. Data on self-reported physician diagnoses of diabetes and hypertension and relevant treatment details were obtained. Systolic and diastolic blood pressure (BP) was measured to the nearest 2 mm Hg using a mercurial sphygmomanometer (Diamond Deluxe BP apparatus, Industrial Electronic and Allied Products) in the right arm supine position.
- *Anthropometric measurements*: Height was measured in centimeters using a standard stadiometer with the participant standing. Weight was measured in kilograms using a body composition monitor (Tanita InterScan BC-601). Body Mass Index (BMI) was calculated by dividing the weight (in kilograms) by the square of the height (in meters).
- *Depression assessment*: Depression was assessed using the Geriatric Depression Scale (GDS-30) (37), administered in the local language by trained clinicians or nurses well-versed in the local language and culture. GDS-30 is a self-reported scale that has been validated extensively. It comprises 30 ‘yes or no’ questions; for questions 1, 5, 7, 9, 15, 19, 21, 27, 29, and 30, a ‘no’ response is scored as one point, and for other questions, a ‘yes’ answer is scored as one. The total score is the sum of the scores of individual questions (maximum score of 30).
- *Blood biochemical tests*: Periodic blood collection camps were organized in the respective villages where the participants were recruited from (given the difficulty in participants coming to the laboratory due to poor public transport facilities in the area). A total volume of 15 ml of peripheral venous blood was collected from each participant under overnight fasting conditions by trained phlebotomists for a detailed panel of biochemical tests that included glucose, triglycerides, high-density lipoprotein (HDL), and low-density lipoprotein (LDL). Glucose estimation was done using the hexokinase method, whereas the enzymatic method was used for lipid parameters.
- *Diagnostic criteria for risk factors*: Diagnoses of the studied conditions/risk factors, namely diabetes, hypertension, obesity, abnormal lipid profile, and depression, were made using the criteria listed in Table 1.

**Table 1:** Diagnostic criteria for risk factors.

Risk factor	Criteria for diagnosis
Hypertension	• Self-reported past diagnosis of hypertension
	• In participants who did not have/were not aware of a past diagnosis, systolic BP ≥ 140 and/or diastolic BP ≥ 90 mmHg
Diabetes	• Self-reported past diagnosis of diabetes
	• In participants who did not have/were not aware of a past diagnosis, fasting blood glucose ≥ 126 mg/dl
Obesity	• BMI ≥ 25 kg/m2 (Asia-Pacific classification)
Abnormal total cholesterol	• Fasting serum total cholesterol > 200 mg/dl
Abnormal triglycerides	• Fasting serum triglycerides ≥ 150 mg/dl
Abnormal HDL	• Fasting serum HDL < 40 mg/dl
Abnormal LDL	• Fasting serum LDL > 100 mg/dl
Depression	• Geriatric Depression Scale (GDS-30) score ≥ 10

This table describes the diagnostic criteria for all the risk factors studied in this manuscript.

BP = blood pressure, BMI = body mass index, HbA1c = hemoglobin A1c (indicates average blood sugar level over the last 3 months), HDL = high-density lipoprotein, and LDL = low-density lipoprotein.

### 2.7. Statistical analysis

All variables were compared between the three periods, namely, the pre-COVID, the COVID, and the post-COVID periods. Categorical variables were checked for statistical association using a Chi-squared test, and the continuous variables were first checked for normality using the Shapiro–Wilk test. Then, as appropriate, an analysis of variance (ANOVA) or Kruskal–Wallis *H*-test was used. A value of *p* < 0.05 was considered statistically significant. A binary logistic regression model for the dichotomous outcome variable (normal = 0, modifiable risk factor present = 1) was adopted to obtain odds ratios (OR) and its 95% confidence interval (CI) for the COVID period and the post-COVID period when compared with the pre-COVID period. The odds ratios were adjusted for marital status, occupation, income, and years of education. All analyses for data were computed using the Statistical Package for Social Sciences (SPSS) software version 26 (IBM Corp, NY, United States).

## 3. Results

Out of the total of 3,148 participants, 1,658 (males: 776; 46.8%, females: 882; 53.2%) belonged to the pre-COVID period, 840 (males: 408; 48.6%, females: 432; 51.4%) belonged to the COVID period, and 650 (males: 308; 47.4%, females: 342; 52.6%) belonged to the post-COVID period, as shown in Table 2. This gender distribution across the three COVID periods was not statistically significant (value of *p* = 0.705). The mean (standard deviation, SD) age of participants in pre-COVID, COVID, and post-COVID periods was 58.3 (10.3) years, 59.0 (9.6) years, and 58.3 (9.4) years, respectively, and this age difference between the three periods was not statistically significant (value of *p* = 0.099). However, the mean (SD) years of education (formal education) was statistically significant (value of *p* < 0.001) between pre-COVID [3.9 (4.6) years], COVID [4.7 (4.6) years], and post-COVID [4.8 (4.8) years] periods. The majority of the study participants were currently married (pre-COVID 77.2%, COVID 81.5%, and post-COVID 84.0%), had an annual income of less than 1 lakh (pre-COVID 96.3%, COVID 93.4%, and post-COVID 97.8%), and were agricultural laborers (pre-COVID 61.7%, COVID 71.8%, post-COVID 71.9%).

**Table 2:** Sociodemographic characteristics of the study participants.

Characteristics	Pre-COVID period (*n* = 1,658)	COVID period (*n* = 840)	Post-COVID period (*n* = 650)	Value of *p*
Age in years, mean (SD)	58.3 (10.3)	59.0 (9.6)	58.3 (9.4)	0.099
Age-group, *n* (%)				
< 65 years	1,142 (68.9)	558 (66.4)	454 (69.8)	0.314
≥ 65 years	516 (31.1)	282 (33.6)	196 (30.2)	
Gender, *n* (%)				
Male	776 (46.8)	408 (48.6)	308 (47.4)	0.705
Female	882 (53.2)	432 (51.4)	342 (52.6)	
Marital status, *n* (%)				
Currently married	1,278 (77.2)	685 (81.5)	546 (84.0)	< 0.001
Others	378 (22.8)	155 (18.5)	104 (16.0)	
Years of education, mean (SD)	3.9 (4.6)	4.7 (4.6)	4.8 (4.8)	< 0.001
Annual income, *n* (%)				
< 1 lakh	1,579 (96.3)	783 (93.4)	630 (97.8)	< 0.001
≥ 1 lakh	60 (3.7)	55 (6.6)	14 (2.2)	
Occupation, *n* (%)				
Agriculture	1,023 (61.7)	587 (71.8)	456 (71.9)	< 0.001
Non-agriculture	634 (38.3)	230 (28.2)	178 (28.1)	

SD, standard deviation.

A few variables used in this study had some missing data. A detailed description of missing values of the variables used in this analysis is shown in Table 3. The reason for missing values included participants’ refusal due to time constraints, data entry errors, and technical problems with equipment. For most of the variables the percentage of missing values was under 10%, and hence, we used pair-wise deletion in the analyses.

**Table 3:** Numbers and percentages of participants with missing data.

Variables	Number	Percentage
Age	0	0
Gender	0	0
Marital status	0	0
Years of Education	245	7.8
Income	27	0.9
Occupation	40	1.3
Hypertension	0	0
Diabetes	0	0
Obesity	412	13.1
Abnormal total cholesterol	251	8.0
Abnormal triglycerides	252	8.0
Abnormal LDL	286	9.1
Abnormal HDL	251	8.0
Depression	326	10.4

This table depicts the numbers and percentages of participants with missing data for all the variables studied in this study.

HDL = high-density lipoprotein and LDL = low-density lipoprotein.

The results of binary logistic regression show that though the odds of hypertension among individuals in the COVID period did not change when compared to the pre-COVID period [OR 0.91, 95% CI (0.75, 1.10)], it increased significantly in the post-COVID period by 1.27 times [OR 1.27, 95% CI (1.04, 1.55)], as shown in Table 4. The odds of diabetes did not differ significantly in COVID [OR 1.22, 95% CI (0.97, 1.52)] and post-COVID period [OR 0.80, 95% CI (0.62, 1.05)].

**Table 4:** Results of binary logistics regression: comparison between the pre-COVID, COVID, and the post-COVID periods.

Outcome variables	Factor	Adjusted ORs (95% CI)	Value of *p*
Hypertension	Pre-COVID period^®^	1	
	COVID period	0.91 (0.75, 1.10)	0.333
	Post-COVID period	1.27 (1.04, 1.55)	0.022
Diabetes	Pre-COVID period^®^	1	
	COVID period	1.22 (0.97, 1.52)	0.088
	Post-COVID period	0.80 (0.62, 1.05)	0.102
Abnormal total cholesterol	Pre-COVID period^®^	1	
	COVID period	0.98 (0.799, 1.20)	0.846
	Post-COVID period	1.58 (1.28, 1.96)	< 0.001
Abnormal triglycerides	Pre-COVID period^®^	1	
	COVID period	1.38 (1.15, 1.66)	0.001
	Post-COVID period	1.23 (1.00, 1.50)	0.050
Abnormal HDL	Pre-COVID period^®^	1	
	COVID period	0.98 (0.81, 1.18)	0.803
	Post-COVID period	0.72 (0.59, 0.88)	0.002
Abnormal LDL	Pre-COVID period^®^	1	
	COVID period	0.92 (0.77, 1.11)	0.371
	Post-COVID period	1.95 (1.57, 2.42)	< 0.001
Obesity	Pre-COVID period^®^	1	
	COVID period	1.42 (1.17, 1.73)	< 0.001
	Post-COVID period	1.32 (1.07, 1.63)	0.010
Depression	Pre-COVID period^®^	1	
	COVID period	2.65 (2.05, 3.44)	< 0.001
	Post-COVID period	3.05 (2.33, 3.99)	< 0.001

This table shows the binary logistic regression model results for the dichotomous outcome variable (normal = 0, modifiable risk factor present = 1) for the COVID and post-COVID periods compared with the pre-COVID period, adjusted with marital status, occupation, income, and years of education.

^®^ = reference group; OR – Odds ratio; CI – Confidence interval.

The odds of obesity among individuals in the COVID period were 1.42 times [OR 1.42; 95% (CI 1.17–1.73)] more than in the pre-COVID period. Similarly, the odds of obesity in the post-COVID period were 1.32 times [OR 1.32; 95% CI (1.07–1.63)] more than in the pre-COVID period.

Further, the odds of abnormal triglycerides were 1.38 times [OR 1.38; 95% (CI 1.15, 1.66)] more among individuals in the COVID period and 1.23 times [OR 1.32; 95% CI (1.00, 1.50)] more in the post-COVID period as compared to the pre-COVID period. The odds of abnormal total cholesterol [OR 0.98; 95% CI (0.799, 1.20)] in the COVID period did not differ statistically when compared to the pre-COVID period; however, in the post-COVID period, it increased by 1.58 times [OR 1.58; 95% CI (1.28, 1.96)] in comparison with the pre-COVID period. A similar trend was observed for abnormal LDL, wherein the odds in the COVID period did not differ statistically [OR 0.92; 95% CI (0.77, 1.11)] but significantly increased by 1.95 times [OR 1.95; 95% CI (1.57, 2.42)] when compared to the pre-COVID period. Interestingly, concerning abnormal HDL, though the odds among individuals in the COVID period did not differ when compared to the pre-COVID period [OR 0.98, 95% CI (0.81, 1.18)], it decreased in the post-COVID period by 0.72 times [OR 0.72, 95% CI (0.59, 0.88)].

The odds of depression among individuals in the COVID period were 2.65 times [OR 2.65; 95% CI (2.05 to 3.44)] more, and that in the post-COVID period were 3.05 times [OR 3.05; 95% CI (2.33 to 3.99)] more than the odds in the pre-COVID period.

In summary, the odds of the having hypertension, abnormal total cholesterol, abnormal LDL, depression, abnormal triglycerides, and obesity increased in the COVID/post-COVID period as compared to pre-COVID period. However, it should be noted that for hypertension, abnormal total cholesterol and abnormal LDL the odds ratio of the post-COVID period is outside the 95% CI of the OR for the COVID period, suggesting an increase in the post-COIVD period compared to the COVID period.

## 4. Discussion

In this study, we aimed to understand the effect of the COVID-19 pandemic on certain modifiable risk factors of dementia, namely, diabetes, hypertension, obesity, dyslipidemia, and depression in a population of rural individuals aged ≥ 45 years, belonging to a prospective, aging cohort from southern India. We observed that the odds of having obesity, abnormal triglycerides, and depression among individuals in the COVID period and the post-COVID period were higher when compared to that in the pre-COVID period. On the other hand, there were higher odds of having hypertension, abnormal total cholesterol, and abnormal LDL only in the post-COVID period and not in the COVID period; there was no significant difference in the odds of having diabetes between any of the three periods.

Worsening obesity and depression during the COVID and post-COVID periods in our study population could be attributed to a variety of reasons, namely increased stress, decreased physical activity during the lockdown, and unhealthy eating habits (19). In addition, we speculate that the pandemic placed a substantial financial strain on the rural participants from Srinivaspura due to the hampering of transport and sale of their agricultural produce. For the other studied risk factors (hypertension, abnormal total cholesterol, and LDL) that seem to have worsened only during the post-COVID period compared to the pre-COVID period, we speculate that this trend could be due to the delayed effect of the COVID-related restrictions on these parameters.

Prior studies across the world (mainly from urban settings) have shown conflicting evidence on the impact of the pandemic on blood pressure (38–43). This ambiguity could be due to methodological issues such as including participants with wide age ranges (18–60 years), different sampling strategies, and robustness of blood pressure monitoring.

Our finding that there was no change in the proportion of diabetes with respect to the pandemic is in line with the results of a recent multicentric study from Italy—the Glycalock study (44). Conversely, several studies from different countries have reported that persons with type 2 diabetes had worsening glycemic control (45–50). Studies from India have shown both worsening (51, 52) and improvement (53) in glycemic control among persons with diabetes during the lockdown period. However, most of these studies have assessed the effect of COVID lockdowns on glycemic control in the short term, unlike our study, wherein the defined COVID period was relatively more extended (the entire period from the start of the first wave to the end of the second wave), which could be one of the possible reasons why we did not see a significant change in the proportions of persons with diabetes in our subjects. We also need to remember that earlier studies from other parts of the world have shown that the pandemic positively affected lifestyle behaviors in certain groups of individuals (54, 55). The varying effects of the pandemic on different population groups are likely due to socio-cultural factors.

Our finding of an increased proportion of obesity in both the COVID and post-COVID periods could be due to a substantial decrease in physical activity and an increase in sedentary behavior in our rural study population due to the pandemic-related movement and social restrictions. A meta-analysis of 61 studies conducted across American, European, and Asian populations showed that COVID-19 was linked with significant decreases in mobility, walking, and physical activity and increases in sedentary activity (56). The majority of our SANSCOG cohort participants are usually engaged in intensive manual labor as part of their agricultural work. Therefore, the movement restrictions due to the lockdown likely resulted in a considerable decrease in their normal/pre-pandemic level of physical activity. Furthermore, the pandemic could have also limited their intake of healthier foods due to restricted access or the severe economic impact, thus resulting in an increase in the proportion of the readily available and cheaper carbohydrates in their diet. A recent study from a metropolitan city in northern India by Ghosh et al. (17) reported carbohydrate consumption increased by 21% among diabetic patients during the lockdown period. It is also a possibility that the increase in psychological stress and depression (as evidenced in this study) was a factor contributing to increased obesity. Studies from several countries (47, 57–59) have clearly shown that BMI / obesity increased during or after the pandemic, and this phenomenon is referred to as “covibesity” or “double pandemic” (60–62).

In line with our study findings, the negative effect of the pandemic on lipid parameters has been demonstrated by previous studies in other countries (49, 63) as well as India (64); changes in lifestyle and stress during the lockdown are likely explanations for this trend. However, an intriguing finding in our study is that there was a worsening of all serum lipid parameters except HDL in the COVID or post-COVID periods. Interestingly, a previous study from eastern India among urban-dwelling males (64) reported a significant deterioration in total cholesterol and triglycerides after the lockdown. However, HDL did not show any significant change; the same pattern was also seen in another study from Slovenia (65). Further, a systematic review by Ojo et al. (47) on 11 studies, predominantly from urban populations worldwide, showed inconsistent effects of the COVID-19 lockdown on lipid parameters. There could be several reasons for such inconsistent findings, such as variations in dietary patterns (a carbohydrate-rich diet is known to increase triglyceride levels and reduce HDL levels (66)) or levels of physical activity and associated medical comorbidities, such as diabetes mellitus and metabolic syndrome.

Lastly, our finding that depression significantly increased during COVID and post-COVID times is expected since our rural cohort underwent tremendous distress due to the severe economic impact of the pandemic in this rural area. A number of studies from India (24, 67–69) and other parts of the world (67, 70) have demonstrated the negative psychological impact of this pandemic, including the rise in depression. Also, this adverse impact has been reported to be higher in older adults than in the general population (71).

Now, it is crucial to take into consideration that all the above-studied risk factors potentially have a bidirectional relationship with COVID-19, (i) the pandemic appears to have an adverse effect on them, as demonstrated by our findings; and (ii) these risk factors have an adverse influence on COVID-19 susceptibility, morbidity, and mortality (72). Therefore, prompt recognition of the worsening of the above risk factors and early intervention measures can be helpful not only in the short term while the pandemic is ongoing but also in the long term in terms of reducing morbidity due to cardiovascular disease, cerebrovascular disease, and of course, dementia.

Our findings are significant since the studied risk factors for dementia are preventable with increased health awareness, simple lifestyle changes, and community-level public health measures, not only in the pandemic but also beyond that. For example, the *India Hypertension Control Initiative*—a partnership initiative between the Ministry of Health and Family Welfare of the Indian central government, state governments, the Indian Council of Medical Research, and the World Health Organization implemented an adaptive strategy in five Indian states during the COVID-19 lockdown to improve access to anti-hypertensive medication for patients with hypertension by means community-based drug distribution at the primary care level and home delivery through frontline workers.

Strengths of our study include a large sample size and a relatively homogenous population. Such studies from India, particularly on aging adults from rural areas, are scarce. Further, using trained clinicians to conduct in-person medical examinations and the objective measurements for all the studied parameters made the assessments robust. This contrasts with many prior studies, which have relied on self-reported measures using web-based or telephonic surveys. Further, we calculated adjusted odds ratios controlling for occupation, income, marital status, and years of formal education, thus partialing out the potential effect of socio-cultural factors.

Our study has some limitations. Due to the study’s cross-sectional design, we could not compare risk factors in the same group of individuals in the pre-COVID, COVID, and post-COVID periods. This, along with non-random sampling, could have resulted in potential confounders when comparing the three groups of individuals. Additionally, the findings in our rural cohort may not be generalizable to other populations worldwide or other parts of India due to the vast socio-cultural diversity. We did not have reliable data on the COVID infection status of these participants (due to their poor awareness and hesitation in testing). So, the direct effects of the pandemic on these risk factors could not be delineated from its indirect effects. Finally, we limited our study to only specific modifiable risk factors as we had objective and robust data on these parameters.

We advocate the need to plan and implement urgent lifestyle-based intervention measures, such as the FINGERS model (73) as well as psychosocial interventions to mitigate this pandemic’s adverse impact and put preventive measures in place to handle similar situations in the future. However, it is essential that these interventions should be tailored according to the needs and acceptability of the Indian population and should also be easily implementable through cost-effective public health measures.

## Data availability statement

The raw data supporting the conclusions of this article will be made available by the authors, without undue reservation.

## Ethics statement

The studies involving human participants were reviewed and approved by Institutional Ethics Committee, Centre for Brain Research. The patients/participants provided their written informed consent to participate in this study.

## Author contributions

JS, AM, PM and VR have made a substantial intellectual contribution to the conception, design, or conduct of the study, and had full access to all the data in the study. JS and AM: acquisition of data. JS, AM, PM and VR: analysis and interpretation of data. JS, AM, PM and VR: drafting and reviewing the manuscript. All authors approved the final version of the manuscript for publication and accept responsibility to submit for publication.

## SANSCOG Collaborators

B.N Gangadhar, Psychiatry, National Institute of Mental Health and NeuroSciences, Bangalore, India, kalyanybg@yahoo.com; Girish N. Rao, Public Health, National Institute of Mental Health and NeuroSciences, Bangalore, India, girishnrao@yahoo.com; Naren P. Rao, Psychiatry, National Institute of Mental Health and NeuroSciences, Bangalore, India, docnaren@gmail.com; Palanimuthu T. Sivakumar, Psychiatry, National Institute of Mental Health and NeuroSciences, Bangalore, India, sivakumar.nimhans@gmail.com.

## Funding

SANSCOG study is funded through the Centre for Brain Research by Pratiksha Trust, the philanthropic arm of Kris Gopalakrishnan and Sudha Gopalakrishnan.

## Conflict of interest

The authors declare that the research was conducted in the absence of any commercial or financial relationships that could be construed as a potential conflict of interest.

## Publisher’s note

All claims expressed in this article are solely those of the authors and do not necessarily represent those of their affiliated organizations, or those of the publisher, the editors and the reviewers. Any product that may be evaluated in this article, or claim that may be made by its manufacturer, is not guaranteed or endorsed by the publisher.
